# Correlation between TM joint disease and rheumatic diseases detected on bone scintigraphy and clinical factors

**DOI:** 10.1038/s41598-020-60804-x

**Published:** 2020-03-11

**Authors:** Ji Suk Shim, Chulhan Kim, Jae Jun Ryu, Sung Jae Choi

**Affiliations:** 1 Department of Dentistry, Korea University Guro Hospital, Seoul, Republic of Korea; 2 Department of Nuclear Medicine, Korea University Ansan Hospital, Gyeonggi-do, Republic of Korea; 3 Department of Dentistry, Korea University Anam Hospital, Seoul, Republic of Korea; 4 Division of Rheumatology, Department of Internal Medicine, Korea University Ansan Hospital, Gyeonggi-do, Republic of Korea

**Keywords:** Health care, Dentistry, Rheumatic diseases

## Abstract

The aim of this study was to evaluate the effect of rheumatic disease as a risk factor for temporomandibular disease (TMD). A total of 143 outpatients reporting symptoms indicating rheumatic disease at their first visit to the rheumatology clinic were included. We evaluated the temporomandibular joint (TMJ) with scintigraphic images, and standard questionnaires were administered for the symptomatic assessment for all patients. The patients were classified into ‘healthy controls’ or as per their diagnosis into ‘osteoarthritis’, ‘axial spondyloarthritis’, ‘peripheral spondyloarthritis’, ‘rheumatoid arthritis’, or ‘other rheumatic diseases’ groups. The patients were also differentiated depending on the presence or absence of axial involvement. The relation between the rheumatic disease type and findings at the TMJ were evaluated using statistical analyses. Axial spondyloarthritis, peripheral spondyloarthritis, and rheumatic arthritis patients showed significantly higher scintigraphic uptake at the TMJ compared with those in the control and osteoarthritis groups (axial spondyloarthritis: 4.5, peripheral spondyloarthritis: 4.5, rheumatoid arthritis: 4.09, control: 3.5, osteoarthritis: 3.4, *p* < 0.0001). Compared with patients without axial involvement, patients with axial involvement also showed significantly higher TMJ scintigraphic uptake (axial involvement: 4.24, without axial involvement: 3.50, *p* < 0.0001) with elevated symptomatic rates in TMD (axial involvement: 17.82, without axial involvement: 9.97, *p* < 0.005).

## Introduction

Rheumatic diseases refer to a group of conditions with a complex immune pathophysiology affecting multiple organ systems^1^. Of these, inflammatory rheumatic arthritic diseases show many systemic abnormalities along with deformities of the synovial structures^2^. The temporomandibular joint (TMJ), as a synovial joint between the temporal bone and the mandible, is also involved in various rheumatic diseases including rheumatoid arthritis (RA)^3,4^, ankylosing spondylitis (AS)^5,6^, and spondyloarthritis (SpA)^7^. In addition, the correlation between laboratory values of various inflammatory markers causing rheumatic diseases and the progression of TMD has been reported. Although the significantly correlated indicators differed depending upon the methods used for evaluating the joint, C-reactive protein (CRP)^8^, rheumatoid factor (RF)^8,9^, erythrocyte sedimentation rate (ESR)^9^, and disease activity score (DAS) 28^10^ showed the correlation with TMJ involvement.

Bone scintigraphy is a nuclear scanning test in which the uptake ratio of radiopharmaceuticals is influenced by the amount of calcium at the phosphate binding sites and the amount of blood flow to the bone^11^. Since detection of inflammatory synovitis of the TMJ is an efficient way to evaluate whether the joint is affected by rheumatic disease^12^, bone scintigraphy can be effectively utilized for the purpose as a sensitive tool for the detection of inflammatory lesions and high osteoblastic activity^13^. Moreover, in investigating the correlation between laboratory indicators and TMD, bone scintigraphy is an ideal method for investigating the TMJ because it is a real-time evaluation^13^, similar to an estimation of the levels of serum inflammatory markers. Both laboratory indices and bone scintigraphy reflect the progression of the inflammatory process, while conventional plain radiographs only show long-standing pathological mineralisation in the bone^14^.

In this study, we aimed to determine the correlation between the TMD and rheumatic diseases. To achieve the purpose, we compared the progress of TMD evaluated with bone scintigraphy and the factors related rheumatic diseases including the category of diseases and the rheumatic markers.

## Methods

### Patients

**Figure 1:**
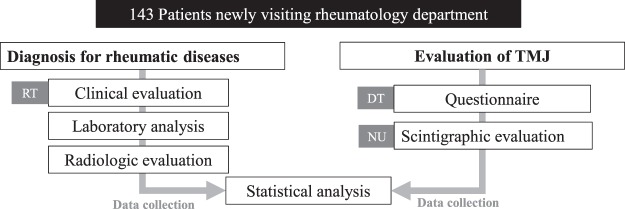
Study design. All recruited subjects were new patients at the rheumatology clinic, and diagnosis of the rheumatic disease and the evaluation of TMJ were processed separately. (RT: Department of rheumatology. NU: Department of nuclear medicine. DT: Department of dentistry).

In this cross-sectional study, we enrolled 143 consecutive patients >18 years of age, who had reported for the first time to the Rheumatology department in Korea University Ansan Hospital between July 2016 and May 2018, with symptoms suggestive of rheumatic disease and were able to comprehend and complete our questionnaires. We did not consider TMJ involvement while selecting the study participants. Patients were excluded if they were undergoing procedures for treatment of TMD or had a history of surgical intervention at the joint. Patients with severe craniofacial anomalies, traumatic TMD, and endocrinal disorders that could affect bone metabolism were excluded as well. This study was approved by the local medical ethics board of Korea University, Ansan Medical Center, Korea (Registration- K2018-0981-002), and the methods were performed in accordance with the relevant guidelines. Written informed consent was obtained from all included patients. All examinations were completed in a week using a double-blind approach to ensure accuracy (Fig. 1).

### Clinical evaluation and laboratory analysis

A single examiner collected all data and diagnostic details of the rheumatic disease, following the diagnosis, in accordance with protocols reported in previous studies^15–17^. Using questionnaires, data on disease duration, number of painful or oedematous joints involved, and presence or absence of positive serum rheumatic factors were collected. An overall assessment of symptom severity in each patient was performed using a visual analogue scale. The mandatory laboratory tests included an assessment of erythrocyte sedimentation rate (ESR), C-reactive protein (CRP), and anti-cyclic citrullinated peptide (anti-CCP) antibody. Rheumatology specialists also performed ancillary studies such as conventional X-ray and ultrasound, when needed. The arthritis was classified as per duration of illness, type of joint involvement, and occurrence of symptoms including morning stiffness and localised swelling or tenderness.

All the diagnoses were made according to the recommended American College of Rheumatology/European League against Rheumatism (ACR/EULAR) classification criteria^15^. With reference to results reported by previous studies that identified the patients as having AS and SpA, in our study, the patients diagnosed with the latter disorder were further divided into axial SpA and peripheral SpA groups. Axial involvement was defined as the occurrence of inflammatory back pain, C1 or C2 vertebral instability, or abnormal axial radiographic features.

### Scintigraphic evaluation of TMJ

**Figure 2:**
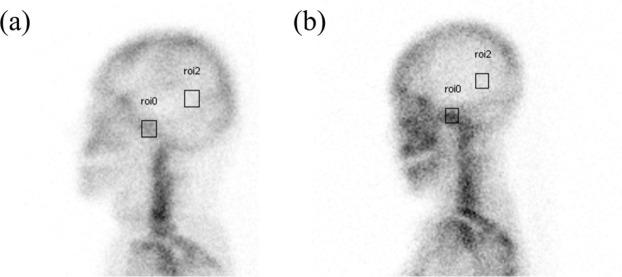
Scintigraphic evaluation. The uptake ratio was acquired by drawing 10 × 10 pixel sized and square-shaped regions of interest (ROIs) on the temporomandibular joint and the parietal area of the skull. The patients were also divided into (**a**) low and (**b**) high scintigraphy uptake groups using the reference uptake value of 3.88 which is a standard value to reveal osteoarthritic results.

The bone scintigraphic images were obtained ~3 hours after an intravenous injection of 740 to 1110 MBq 99mTc-methylene diphosphonate (MDP). In each patient, whole-body images were taken first, and additional regional images including the right and left lateral images of the skull were captured using GE Infinia gamma camera (GE Healthcare, Milwaukee, Wisconsin, U.S.A.). The imaging was performed using a low energy high-resolution (LEHR) collimator with energy window of 20% at 140 keV, and matrix size of 256 × 256. The analysis of the skull images was performed by drawing 10 × 10 pixel sized, square-shaped regions of interest (ROIs) on the temporomandibular joint and the parietal area of the skull (Fig. 2). The uptake ratio of the temporomandibular joint was calculated as the TMJ ROI counts divided by those of the parietal skull. The patients were also divided into high and low scintigraphy uptake groups using the reference uptake value of 3.88 which is a standard value to reveal osteoarthritic results^18^.

### Questionnaire for symptom evaluation of TMD

In order to evaluate the symptoms of TMD, we used the questionnaire proposed by Fonseca with some modifications^19^. It comprised 8 questions evaluating the patients for occurrence of symptoms including pain (involving the TMJ, head, and neck), limitations of mandibular movement, TMJ clicking, discomfort while opening the mouth or chewing, and emotional stress. The patients could select ‘yes’, ‘sometimes’, or ‘no’ as their response to each question, and the answers were scored as 10, 5, or 0, respectively. The expected total score was ranged between 0 to 80 and called symptomatic rates because it reflected the symptomatic severity of TMD.

### Statistical analysis

The categorical variables were expressed as frequency and percentages, while the continuous variables were expressed as mean with standard deviation (SD) or as median with the interquartile range (IQR). We utilised chi-squared test to compare the distribution of categorical variables according to rheumatic diseases or axial involvement. Student’s *t*-test or analysis of variance (ANOVA) were performed to assess the difference of Scintigraphic uptake ratio and symptomatic rate between groups, as appropriate. We conducted post-hoc analysis using Tukey’s honest significant difference (HSD) to evaluate the statistical significance between the study groups. The correlations between temporomandibular joint parameters and rheumatic laboratory indices were evaluated using Pearson’s correlation method and calculated p-value testing the null hypothesis that the correlation is zero.

We utilised Student’s *t*-test, the *chi*-squared test, analysis of variance (ANOVA), and post-hoc analysis using Tukey’s honest significant difference (HSD) to evaluate the statistical significance between the study groups. The correlations were evaluated for statistical significance using Pearson’s coefficient. A *p*-value of < 0.05 was considered statistically significant. All statistical analyses were carried out using SAS software version 9.4 (SAS Institute Inc., Cary, NC, USA).

## Results

### Comparison of bone scintigraphy findings and symptomatic rates between different rheumatic diseases

Table 1 shows the comparisons between patients with OA, those with other rheumatic diseases, and those in the healthy control (HC) group, with respect to factors including age, sex, disease duration, and TMJ parameters (high/low uptake, uptake ratio, symptomatic rate). The patients who showed negative results through examinations were divided into health control. The patients diagnosed with psoriatic arthritis, undifferentiated sacroiliitis, and gout were excluded due to the lack of sufficient numbers required to perform the statistical analysis. All groups had a higher proportion of females except for the axial SpA group which included more men than women. A high uptake on TMJ bone scintigraphy was demonstrated by the maximum number of patients diagnosed with peripheral SpA (77.8%), while the HC group had the lowest number. The patient-groups arranged as per their scintigraphy uptake ratios from the highest to lowest values were peripheral SpA, axial SpA, RA, HC, and OA groups, respectively. Patients with peripheral SpA, axial SpA, and RA showed statistically significant uptake on bone scintigraphy as compared to that of those in the OA and the HC groups (*p* < 0.0001). However, a comparison of symptomatic rates between the groups showed no statistical significance (*p* > 0.05) with respect to a particular group.

**Table 1:** Descriptive statistics and comparison between patients with rheumatic diseases and healthy controls. *p < 0.05 by ANOVA; Groups with the same letter did not show any statistically significant differences (*P* ≥ 0.05) by Tukey’s HSD test. N: number, SD: standard deviation.

*Diseases*	Healthy Control	Osteoarthritis	Rheumatic Arthritis	Axial Spondyloathritis	Peripheral Spondyloathritis
*Total number*	19	56	21	15	18
*Gender*, *N (Male/Female)*	6/13	17/39	5/16	9/6	6/12
*Age (years)*, *Mean (SD)*	42.5 (13.3)	51.6 (10.6)	53.7 (16.3)	39.1 (11.2)	32.4 (9.8)
*Disease duration* *(months), Mean (SD)*	18.5 (20.4)	30.5 (31.2)	51.5 (32.3)	20.6 (19.9)	21.6 (28.9)
**<*TMJ Parameters*>**					
*High uptake* *(*≥*3.88), N* (*%)*	6 (31.6%)	19 (33.9%)	13 (61.9%)	11 (73.3%)	14 (77.8%)
Uptake ratio*, *Mean* (*SD)*	3.5 (0.9)^A^	3.4 (0.8)^A^	4.09 (1.1)^B^	4.5 (1.2)^B^	4.5 (0.9)^B^
Symptomatic rate*, *Mean* (*SD)*	13.0 (12.5)^A^	12.4 (16.2)^A^	10.4 (12.9)^A^	8.08 (11.5)^A^	21.8 (26.1)^A^

### Association of bone scintigraphic findings and symptomatic rates with axial involvement

Table 2 shows a comparison of the uptake ratios and the symptomatic rates between patients with and without axial involvement. Patients with axial involvement demonstrated a higher uptake ratio (4.24) on scintigraphy as compared to patients with no evidence of axial involvement (3.5) with the difference being statistically significant (*p* < 0.0001). The rate of occurrence of symptoms also showed a statistically significant difference (*p* < 0.005) between patients with axial involvement (17.82), who were more symptomatic than those without (9.97).

**Table 2:** Scintigraphic uptake ratio and symptomatic rate depending on axial involvement.

	Control (do not involve axial articular, N = 81)	Axial involvement (N = 48)	P*
***Uptake ratio***			< **0.0001**
*Mean (SD)*	3.50 (0.89)	4.24 (1.03)	
*Median (Q1, Q3)*	3.27 (2.89, 4.10)	4.09 (3.52, 4.83)	
*(Min, Max)*	(1.76, 6.10)	(2.22, 6.89)	
***Symptomatic rate***			< **0.05**
*Mean (SD)*	9.97(12.05)	17.82 (20.89)	
*Median (Q1, Q3)*	7.50 (0.00, 20.00)	10.00 (0.00, 30.00)	
*(Min, Max)*	(0.00, 40.00)	(0.00, 75.00)	

*P by Student’s t-test. N: number, SD: standard deviation, Q1: quartile 1 Q3: quartile 3.

### Association between symptoms and scintigraphic findings

The correlation between the symptomatic rate and the scintigraphic uptake ratio was 0.10 with a *p*-value of 0.19, which was not statistically significant.

### Correlation between laboratory indices and TMJ parameters

Table 3 shows the correlation between the patients’ laboratory indices and the TMJ parameters. Inflammatory markers characteristic of inflammation in rheumatic diseases including ESR and CRP were found to show a statistically significant correlation (*p* < 0.0005 and *p* < 0.005, respectively) with the scintigraphic uptake ratio, while showing a correlation coefficient value. Table 4 shows the comparison of the levels of rheumatic markers between the high- and low-scintigraphic uptake patient groups. The patients demonstrating high scintigraphic uptake showed higher ESR and CRP values as compared to the patients with low scintigraphic uptake with the difference being statistically significant (*p* < 0.05).

**Table 3:** Correlation coefficient and the level of significance between temporomandibular joint parameters and rheumatic laboratory indices.

	ESR		CRP		Anti-CCP		Tender (number)	
	**CC**	P*	**CC**	P*	**CC**	P*	**CC**	P*
*Uptake ratio*	0.18	**<0.005**	0.18	**<0.005**	−0.05	**0.46**	−0.04	**0.54**
*Symptomatic rate*	−0.08	**0.31**	0.03	**0.66**	0.05	**0.50**	0.04	**0.57**
	**Swollen (number)**		**DAS28 (ESR)**		**DAS28 (CRP)**		**Disease Duration**	
	**CC**	P*	**CC**	P*	**CC**	P*	**CC**	P*
*Uptake ratio*	−0.10	**0.12**	0.03	**0.66**	0.03	**0.66**	−0.04	**0.52**
*Symptomatic rate*	0.02	**0.77**	−0.03	**0.73**	0.04	**0.61**	−0.05	**0.53**

*P for null hypothesis that correlation is zero by Pearson’s correlation method. ESR: erythrocyte sedimentation rate, CRS: C-reactive protein, RF: rheumatoid factor, CC: Pearson’s correlation coefficients.

**Table 4:** Comparison of rheumatic indicators between high and low uptake groups.

	ESR	CRP	Anti-CCP	Tender (number)
High uptake (≥3.88) Mean(SD)	26.75 (23.97)	0.91 (2.07)	34.24 (166.7)	8.21 (7.69)
Low uptake (<3.88) Mean(SD)	17.56 (20.72)	0.32 (0.64)	40.10 (180.5)	8.65 (7.33)
P*	**<0.05**	**<0.05**	**0.85**	**0.74**
	**Swollen (number)**	**DAS28** (**ESR)**	**DAS28** (**CRP)**	**Disease Duration**
*High uptake (*≥*3.88)* Mean(SD)	1.38 (2.62)	3.59 (1.08)	3.09 (1.02)	20.19 (27.40)
*Low uptake (*<*3.88)* Mean (SD)	1.63 (2.60)	3.43 (0.95)	3.04 (0.88)	23.27 (29.88)
P*	**0.58**	**0.38**	**0.79**	**0.81**

ESR: erythrocyte sedimentation rate, *P by Student’s t-test. CRS: C-reactive protein, RF: rheumatoid factor, CC: correlation coefficients.

### Correlation between age and TMJ parameters

Table 5 shows the correlation between patient age and TMJ parameters. The statistical analysis of the total population in this study showed that the patient age correlated significantly with the scintigraphic uptake ratios (*p* < 0.001). The HCs and the OA patients also showed a statistically significant correlation between age and scintigraphic uptake ratios (*p* < 0.05), but no significant correlation of age was observed in patients with peripheral SpA, axial SpA, and RA (*p* > 0.05).

**Table 5:** Correlation of age with scintigraphic uptake and symptomatic rate.

	Total patients	HC and OA	inflammatory rheumatic diseases (RA, axial SpA, and peripheral SpA)
***Uptake***			
CC	−0.44	−0.48	−0.26
P*	**<0.0001**	**<0.05**	**0.26**
***Symptomatic rate***			
CC	−0.13	−0.12	0.34
P*	**0.20**	**0.68**	**0.27**

*P for null hypothesis that correlation is zero by Pearson’s correlation method. HC: healthy control, OA: osteoarthritis, RA: rheumatic arthritis, SpA: spondyloarthritis, N: number, SD: standard deviation, CC: correlation coefficients.

## Discussion

Arthritis of the TMJ progresses through the serial processes of inflammation causing bony changes with articular surface remodelling, narrowing of the joint space, cortical thickening, and the development of osteophytes. Furthermore, TMJ arthritis associated with rheumatic diseases can progress to inflammatory joint destruction, for which a surgical reconstruction of the affected condyle may be necessary^20^. Therefore, identifying rheumatic disease patients at risk of TMJ arthritis is clinically important, so that the disease progression can be controlled from an early stage with appropriately applied therapeutic strategies^21,22^. As bone scintigraphy can detect inflammation, it is an appropriate imaging modality to evaluate a symptomatic TMJ in rheumatic disease patients, because conventional radiography does not show the early pre-resorptive stage of joint inflammation^21^. Bone scintigraphy has a high sensitivity for detecting bone resorption as it can detect early changes in the bone structure that occur subsequent to only a ~10% increase in osteoblastic activity^23–25^. Furthermore, bone scintigraphy helps provide quantitative data^26^ and operator-independent results as compared to those provided by conventional radiography^18^. In this study, we used bone scintigraphy to evaluate the effect of rheumatic diseases on the TMJ, and our results demonstrate that the patient suffering from peripheral SpA, axial SpA and RA are at a high risk for developing TMJ arthritis. We also found that TMJ arthritis is significantly likely to occur in patients with rheumatic arthritis involving the axial structures.

The aetiology of TMD is multifactorial in nature^27,28^, which are further classified into three types as predisposing, initiating, and perpetuating factors^27,29^. The predisposing factors enhance the risk of development of TMD, initiating factors lead to a start of the symptoms, and perpetuating factors either interrupt the healing process or prompt a progression of the disease^14,30^. Rheumatic disease is categorised as a predisposing factor that causes an inflammation of the synovial structures of the TMJ^14^. In this study, patients with RA, peripheral SpA, and axial SpA showed significantly higher scintigraphic uptake ratios as compared to those in the other groups, and the results suggest that these disorders may have a greater association with TMD-predisposing factors. In addition, though the patients with these disorders may have no present symptoms indicating TMJ involvement, they should be cautioned to avoid exposure to the initiating risk factors including occlusal interference, oral parafunction, and trauma.

Interestingly, the results of this study also showed that an axial involvement is associated with a significantly higher scintigraphic uptake ratio at the TMJ as well as with a higher symptomatic rate. No previous study has evaluated the correlation between axial involvement in rheumatic diseases and TMD, though the results of other studies indirectly support this association. AS is a characteristic rheumatic disease that primarily affects the axial skeleton^31^ and in which, the TMJ is involved at an early stage^32^. In contrast, as the axial skeleton is frequently affected only in long-standing and severe RA^33,34^, TMJ involvement is usually found at a later and advanced stage of the disease^8,9^. Thus, it can be surmised that TMJ involvement in patients suffering from rheumatic disease corresponds to the duration of involvement of the axial joints. Previous studies also show that changes in body posture that occur secondary to involvement of axial joints, can act as an initiating factor for TMD. There is a definite correlation between posture and structural changes in the stomatognathic system^35^. Changes in mandibular position are induced by variations in body posture, subsequently affecting the condylar position and the tension of the muscles supporting the mandible^36^. Therefore, previous studies have shown how patients with TMD presented with anteriorly positioned heads^37,38^ and also how postural changes in the cervical region can cause TMD^39^.

Determining which laboratory indices have a correlation with TMD is necessary to utilise them as diagnostic tools. In this study, the patients showing high scintigraphic uptake (≥3.88) at the TMJ, which indicated a high possibility of existing TMD^18^, showed significantly higher ESR and CRP levels as compared to the patients demonstrating low scintigraphic uptake ( < 3.88). However, the levels of inflammatory markers were not found to correlate significantly with the TMJ parameters in this study. ESR and CRP are non-etiologic, non-specific inflammatory markers, which when elevated, simply indicate the presence of inflammation in the body. The low correlation of these indices with TMJ parameters inevitably occurs, because high levels of ESR and CRP can occur secondary to any arthritis in the body. However, the high ESR and CRP levels that were found in patients showing high scintigraphic uptake suggest that these laboratory indices can be utilised as indicators of progressive inflammation of the TMJ.

This study showed no correlation between the scintigraphic uptake ratios and the symptomatic rates of TMD. Previous studies showed similar results, which indicate that the discrete symptoms were a result of the synovial inflammatory process leading to structural changes in the TMJ^9,40^. TMJ inflammation by itself is hardly symptomatic, because the characteristic structure of the retrodiscal tissue of the joint is rich in blood vessels, which contributes to a resorption of exudates^9^. Moreover, rheumatic diseases play a definite role as predisposing factors for TMD^14^, which means that additional initiating factors are still necessary to cause progression and symptomatic manifestations. That RA along with axial and peripheral SpA did not have significantly increased symptomatic rates in spite of the associated significantly high scintigraphic uptake ratios, also supports this theory.

Aging is a well-known factor that has a correlation with TMD^41^. This study also shows that age correlates negatively with scintigraphic uptake ratios for the total study population, indicating that younger patients are at a higher risk of TMJ inflammation. Interestingly, however, the correlation was different between patients with and without inflammatory rheumatic disease. In contrast to the HCs and OA patients, patients with inflammatory rheumatic diseases showed no correlation between scintigraphic uptake ratio and age. This result suggests that the inflammation of TMJ in inflammatory rheumatic disease patients correlates with the severity of the disease and with axial involvement rather than with their age.

The method of sampling patients critically affects the results of clinical studies. In this study, the subjects of this study were patients with suspected rheumatic disease, regardless of TMJ involvement. Although the overall symptomatic rates of TMD in this report are relatively lower than those in previously reported studies owing to the study design, patients with all types of rheumatic disorders underwent TMJ evaluation. Therefore, we could evaluate the effect of various rheumatic diseases on the TMJ as compared to previous studies. The time interval between investigation and diagnosis is also important in clinical studies. Longer duration between each examination and delay in diagnosis may give rise to skewed correlations between the disease and the factors under investigation. Moreover, inclusion of patients under treatment also affects the results as the inflammatory indicators and scintigraphic uptake ratios can easily improve with continued management. To acquire more accurate results, we only included new, heretofore untreated patients at their first visit to our centre in this study and all patient examinations were completed within a week. However, the limitation of this study is that some rheumatic diseases, which may affect TMD such as psoriatic arthritis, undifferentiated sacroiliitis, and gout, were excluded because of the small number of patients. Further studies according to the relationship between the excluded rheumatic diseases and TMD are recommended.

In conclusion, our results underline that axial SpA, peripheral SpA, and RA can act as TMD-predisposing factors causing inflammation in TMJ and that axially involved arthritis is a critical reason of developing TMD in rheumatic disease patients. In addition, ESR and CRP can be utilized as indicators of progressive inflammation of the TMJ.
